# IDENTIFYING FACTORS THAT CONTRIBUTE TO THE FINANCIAL STRAIN OF HISPANIC CAREGIVERS OF OLDER ADULTS

**DOI:** 10.1093/geroni/igad104.3405

**Published:** 2023-12-21

**Authors:** Margret Howard, Wingyun Mak, Orah Burack

**Affiliations:** The New Jewish Home Research Institute on Aging, New York City, New York, United States; The New Jewish Home Research Institute on Aging, New York City, New York, United States; The New Jewish Home Research Institute on Aging, New York City, New York, United States

## Abstract

Informal caregiving has been shown to have negative effects on the caregiver’s physical, mental, and financial health. To date, studies have mainly focused on the physical and mental health of caregivers; however, finances too are an area of caregiver vulnerability. In this study we examined which types of financial hardships are the greatest predictors of financial strain in Hispanic caregivers (N=184) for persons 50 or older. Data were obtained from the American Association of Retired Persons (AARP) Caregiving in 2020 survey, which oversampled for Hispanic caregivers. A hierarchical regression analysis was conducted to predict caregiver financial strain. Independent variables were entered in three steps: (1) age and gender, (2) emotional strain, physical strain, length of expected caregiving, and attitude towards caregiving (3) taking on more debt, borrowing money, unable to afford basic expenses, late/unpaid bills, using short-term savings, using long-term savings, being unable to save, and moving to a less expensive home. Age and gender were not significant (ΔR2=.007, p=.531). Emotional (β=.309, t=3.512, p<.001), and physical strain (β=.229, t=2.627, p=.009) were both predictors of financial strain (ΔR2=.312, p<.001). Additionally, the specific financial hardships related to financial stress above and beyond steps one and two (ΔR2=.137, p<.001) were being unable to afford basic expenses (β=.356, t=4.161, p<.001), and using long term savings (β=-.308, t=-4.372, p<.001). These results have implications for education and interventions concerning financial hardships and options for Hispanic caregivers of older adults.

